# Supplementary material for: Angelica sinensis polysaccharide as potential protectants against recurrent spontaneous abortion: focus on autophagy regulation
Source: Front Med (Lausanne). 2025 Jan 15;12:1522503. doi: 10.3389/fmed.2025.1522503 (PMC11774876; doi:10.3389/fmed.2025.1522503)
Supplement: Supplementary material S1 — The main instruments used during the LC-MS process, along with their models/specifications and manufacturers. [file Supplementary_file_1.zip › Metabolomics sequencing data FC1.2/6.差异代谢物/volcano/Exposure_Ctrl.pdf]

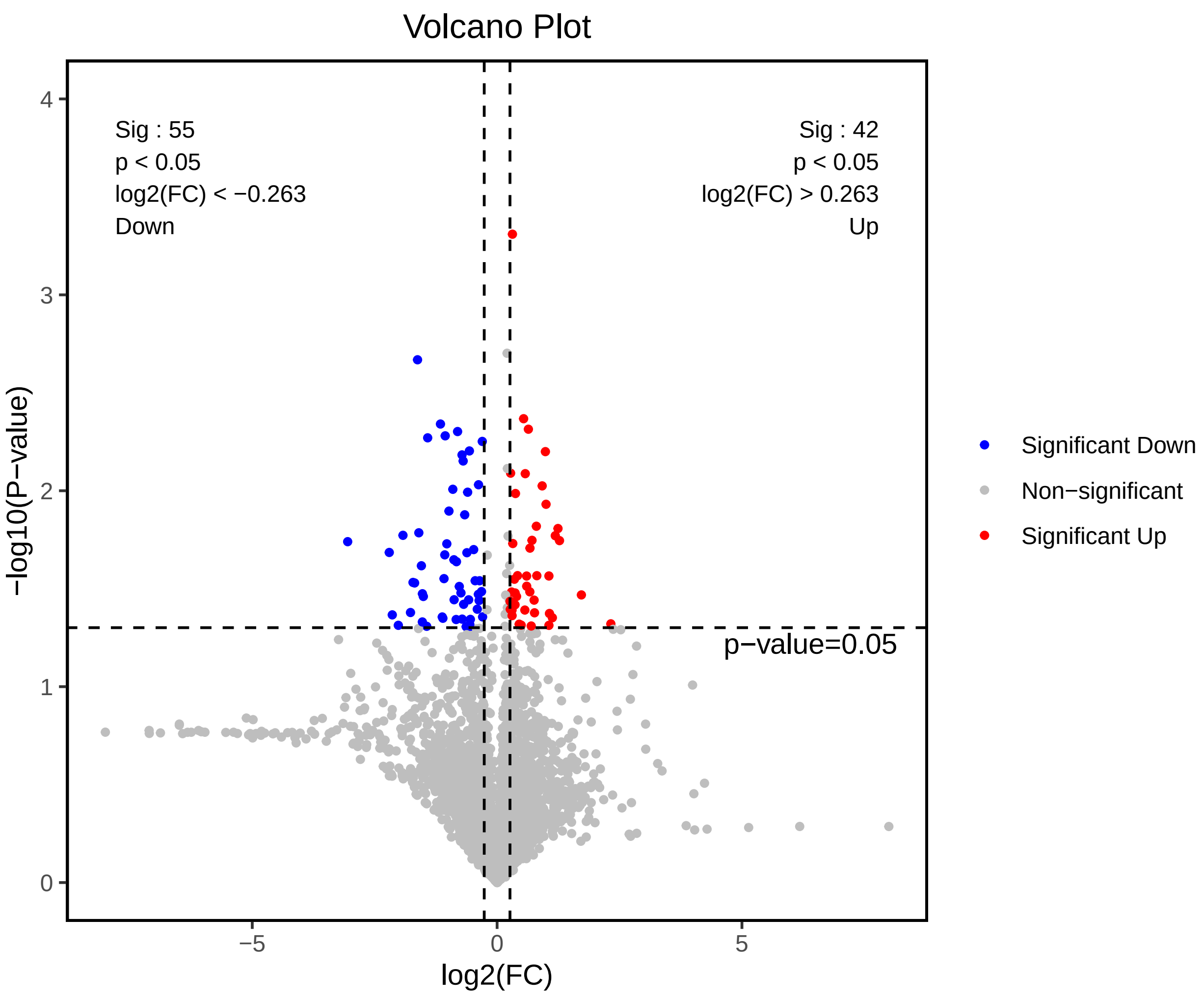

Volcano Plot
Sig : 55
p < 0.05
log2(FC) < −0.263
Down
Sig : 42
p < 0.05
log2(FC) > 0.263
Up
p−value=0.05
−log10(P−value)
log2(FC)
0
1
2
3
4
−5
0
5
Significant Down
Non−significant
Significant Up
